# Supplementary material for: Harnessing Inflammatory Monocytes to Overcome Resistance to Anti-PD-1 Immunotherapy
Source: bioRxiv. 2026 Feb 8:2026.02.05.704029. Preprint. [Version 1] doi: 10.64898/2026.02.05.704029 (PMC12889677; doi:10.64898/2026.02.05.704029)
Supplement: Supplement 1 [file media-1.pdf]

Supplementary Table 1

Inflammatory_monocytes	Immunosuppressive_macrophages
Ly6i	Mmp12
AA467197	Fabp5
Prdx5	Fabp4
Cfb	Gpnmb
Sod2	Ctsl
Lyz2	Ftl1
Clec4e	Lgals3
Nos2	Fth1
C3	Atp6v0d2
Clec4n	Gdf15
Saa3	Prdx1
H2-Ab1	Hmox1
H2-Eb1	Mmp13
Cd74	Esd
Upp1	Cd63
Cxcl9	Spp1
Tgfbi	Gpr137b
Fpr2	Cd68
Fbxl5	Ndrg1
H2-Aa	Lpl
Pla2g7	Ctsb
Ctsc	Cd36
Ly6a	Pmp22
Ass1	Dnmt3a
Slc7a11	Il1rn
Acod1	Trem2
Cxcl10	Atp6v1a
Cybb	Cstb
Ifi30	Hilpda
Lyz1	Pgam1
Gbp2	Aldoa
Mcemp1	Ctsd
Il1b	Creg1
Ifitm3	Basp1
Cd14	Anxa1
Bst1	Bnip3
Ptgs2	Mt1
Aif1	Lipa
Plac8	Plin2
H2-DMb1	Gde1
Clec12a	Igf1

Blvrb	Mif
Marcksl1	Osbpl8
Tspo	Samd8
Slamf8	Bhlhe41
Pilra	Timp2
Cxcl2	Serpinb6a
Iigp1	Psmb6
Klra2	Plk2
H2-DMa	Adam8
Pid1	Slc2a1
Atox1	Clec4d
Clec5a	Psmd8
Ccrl2	Akr1a1
Slc7a2	Atp6v0c
Cxcl16	Tmem65
Prdx6	Ccl8
Nampt	Vat1
Pirb	Tpi1
Capg	Ero1l
Cstb	Plau
Calhm6	Card19
Gm15056	Ldha
Glrx	Ninj1
Slc16a3	Abca1
Grina	Atp6v1c1
Txn1	Ctsz
Magohb	Gapdh
Cebpb	Rnh1
Tgm2	Pkm
Ms4a6c	Cd9
Cyba	Lhfpl2
Zbp1	Gng11
F10	Gclm
S100a11	Ctsk
Gda	Eif4ebp1
Clec4d	C1qc
Bcl2a1a	Fam129b
Slpi	Il7r
Lpcat2	Ctsa
Acsl1	Vim
Psme2	C1qb
Lst1	Rilpl2
Dram1	Tmem189

Ms4a4c	Bsg
Prdx1	Sgk1
Pnp	Capg
Tlr2	Gyg
Ehd1	Sat1
Fcer1g	Blvrb
Psmb10	Pgk1
Fth1	Psap
Hck	Atf3
Msr1	Por
Itgb2	Pf4
Ms4a6d	Lrp12
Pkm	Mt2
Ctsz	Nceh1
Bcl2a1b	Hk2
Gpr141	Gstm1
Naaa	Lgmn
Ier3	Nos2
Rab32	Pld3
Inhba	Gabarap
Cfp	Fnip2
Fn1	Rassf8
Msrb1	Fblim1
Cd40	Cpeb4
Samhd1	Bnip3l
Arg1	Mtss1
Mmp14	Vcam1
Vim	Slc30a1
Plaur	Rnf128
Esd	Anxa4
Tnfaip2	Atp6v1g1
Fgr	Sqstm1
Cnih4	Sdcbp
Smpdl3b	Ncoa4
Tyrobp	Srxn1
Atp6v0c	Grn
Tapbp	Litaf
Csf1r	Cyba
Gpx1	Lamp1
Procr	Apoe
Pgd	Ftl1-ps1
Fcgr4	Lgals1
Cdkn1a	Lat2

Psmb8
Itgam
Spi1
Tpi1
Sdc4
Rnf19b
Isg15
Alas1
Alox5ap
Ncf4
Mcub
Tma16
Emp3
Mif
Cd52
Ly6c2
App
Socs3
Nrp2
Ifitm2
Gapdh
Dusp1
Lair1
Gsn
Nlrp3
Psma7
Creb5
Fcgr1
Plbd1
Sh3bgrl3
Snx10
Ninj1
Aldoa
Ptpn6
Gbp4
Nupr1
Stat1
Pomp
Sdc3
Srgn
Ctss
Ggh
Cd274
Slc6a8
Ccl12
Soat1
Tent5c
Hexa
Mgst1
Atp6v0e
Emp1
Pdpn
Gadd45a
Bri3
Sh3glb1
Slc7a2
Cyb5a
Vdac1
Gla
Vegfa
Slamf7
Anpep
Vdac2
Vapa
Slc48a1
Cxcl16
C1qa
Hist1h2bc
Gna13
Rgl1
Syngr1
Ankrd37
Dstn
Atp6v0b
Gng2
Arhgap10
Grb2
Ap3s1
Myo5a
Tpp1
Abcg1
Dhrs3
Npc2
Gpi1
Rgs1
Ccl9

Irf7	0610012G03Rik
Slc11a1	Rcbtb2
Sirpb1c	Mxi1
Mvp	Arl8b
Slc7a8	Anxa5
Ly6e	Rsad2
Fcgr2b	Sdc1
Tppp3	Atp6v1b2
Unc93b1	Slc27a1
N4bp1	Rhoc
Hk3	Mpc1
Ptpn1	Ctss
Fos	Hebp1
Prkcd	Tmem106a
S100a4	Ms4a7
Ncf1	Ugp2
Vamp8	Vcl
Card19	Plekho1
Ifi27l2a	Rnasek
Rrbp1	Aph1c
Ccr1	Plxna1
Eno1	Abcc5
Ltb4r1	Rragc
Cyfip1	Hspa1a
Pgam1	Aprt
Ppt2	Ccl6
BC028528	Rala
Tmbim4	Flrt2
Scimp	C3ar1
Gm4951	Pdgfa
