# Supplementary material for: Harnessing Inflammatory Monocytes to Overcome Resistance to Anti-PD-1 Immunotherapy
Source: bioRxiv. 2026 Feb 8:2026.02.05.704029. Preprint. [Version 1] doi: 10.64898/2026.02.05.704029 (PMC12889677; doi:10.64898/2026.02.05.704029)
Supplement: Supplement 2 [file media-2.pdf]

Supplementary Table 2 Antibodies				
**Target**	**Clone**	**Company**	**Fluorophore**	**Dilution**
Arg1	A1exF5	eBioscience	AF700	1:200
CD11b	M1/70	BD Biosciences	BUV395	1:800
CD11b	M1/70	BioLegend	BV421	1:400
CD11b	M1/70	BioLegend	BV650	1:400
CD19	1D3	BD Biosciences	PE	1:400
CD206	C068C2	BioLegend	AF594	1:50
CD206	C068C2	BioLegend	FITC	1:200
CD206	C068C2	BioLegend	BV421	1:200
CD3ε	145-2C11	BioLegend	BV421	1:100, 1:400
CD4	GK1.5	BioLegend	PerCP/Cy5.5	1:200, 1:400
CD4	GK1.5	BioLegend	FITC	1:400
CD44	IM7	BioLegend	APC/Cy7	1:200
CD45	30-F11	BioLegend	BV785	1:400
CD45	30-F11	BioLegend	PerCP/Cy5.5	1:400
CD45.2	104	BioLegend	BV605	1:400
CD86	GL-1	BioLegend	PE/Cy7	1:400
CD86	GL-1	BioLegend	BV785	1:400
CD8α	53-6.7	BioLegend	AF594	1:200
CD8β	YTS156.7.7	BioLegend	BV510	1:200, 1:400
CD8β	YTS156.7.7	BioLegend	PerCP/Cy5.5	1:200
F4/80	BM8	BioLegend	AF594	1:400
F4/80	BM8	BioLegend	APC/Cy7	1:200
F4/80	BM8	BioLegend	PerCP/Cy5.5	1:200
F4/80	T45-2342	BD Biosciences	BUV563	1:400
H-2Db	KH95	BioLegend	APC	1:100
H-2Kb	AF6-88.5	BioLegend	FITC	1:100
IFNγ	XMG1.2	BioLegend	PE	1:100, 1:400
IL-12/23-p40	C15.6	BioLegend	PE/Dazzle 594	1:100
IL-6	MP5-20F3	eBioscience	FITC	1:100
iNOS	W16030C	BioLegend	PE	1:400
Ly6G	1A8	BioLegend	PE/Cy7	1:400
Ly6C	HK1.4	BioLegend	BV510	1:400
Ly6C	HK1.4	BioLegend	BV605	1:400
Ly6C	HK1.4	BioLegend	FITC	1:400
MHCII	M5/114.15.2	BioLegend	PerCP/Cy5.5	1:400
MHCII	M5/114.15.2	BioLegend	PE	1:400
NK1.1	PK136	BioLegend	AF700	1:400
NK1.1	PK136	BioLegend	PE	1:400
NKG2D	CX5	BioLegend	PE/Dazzle 594	1:200
NKp46	29A1.4	BioLegend	PE/Cy7	1:100, 1:200
PD-1	29F.1A12	Biolegend	BV421	1:200

PD-1	APC	Biolegend	APC	1:200
TCRβ	H57-597	BioLegend	PE	1:400
TCRβ	H57-597	BioLegend	BV785	1:400
TCRβ	H57-597	BioLegend	APC	1:400
TCR$\gamma\delta$	GL3	BioLegend	PE/Cy7	1:400
Tim-3	RMT3-23	BioLegend	BV605	1:100
TNFα	MP6-XT22	BioLegend	BV605	1:100
