# Supplementary material for: Harnessing Inflammatory Monocytes to Overcome Resistance to Anti-PD-1 Immunotherapy
Source: bioRxiv. 2026 Feb 8:2026.02.05.704029. Preprint. [Version 1] doi: 10.64898/2026.02.05.704029 (PMC12889677; doi:10.64898/2026.02.05.704029)
Supplement: 4 [file NIHPP2026.02.05.704029v1-supplement-4.pdf]

Supplementary Figure 1

bioRxiv preprint doi: https://doi.org/10.64898/2026.02.05.704029; this version posted February 8, 2026. The copyright holder for this preprint (which was not certified by peer review) is the author/funder, who has granted bioRxiv a license to display the preprint in perpetuity. It is made available under aCC-BY 4.0 International license.

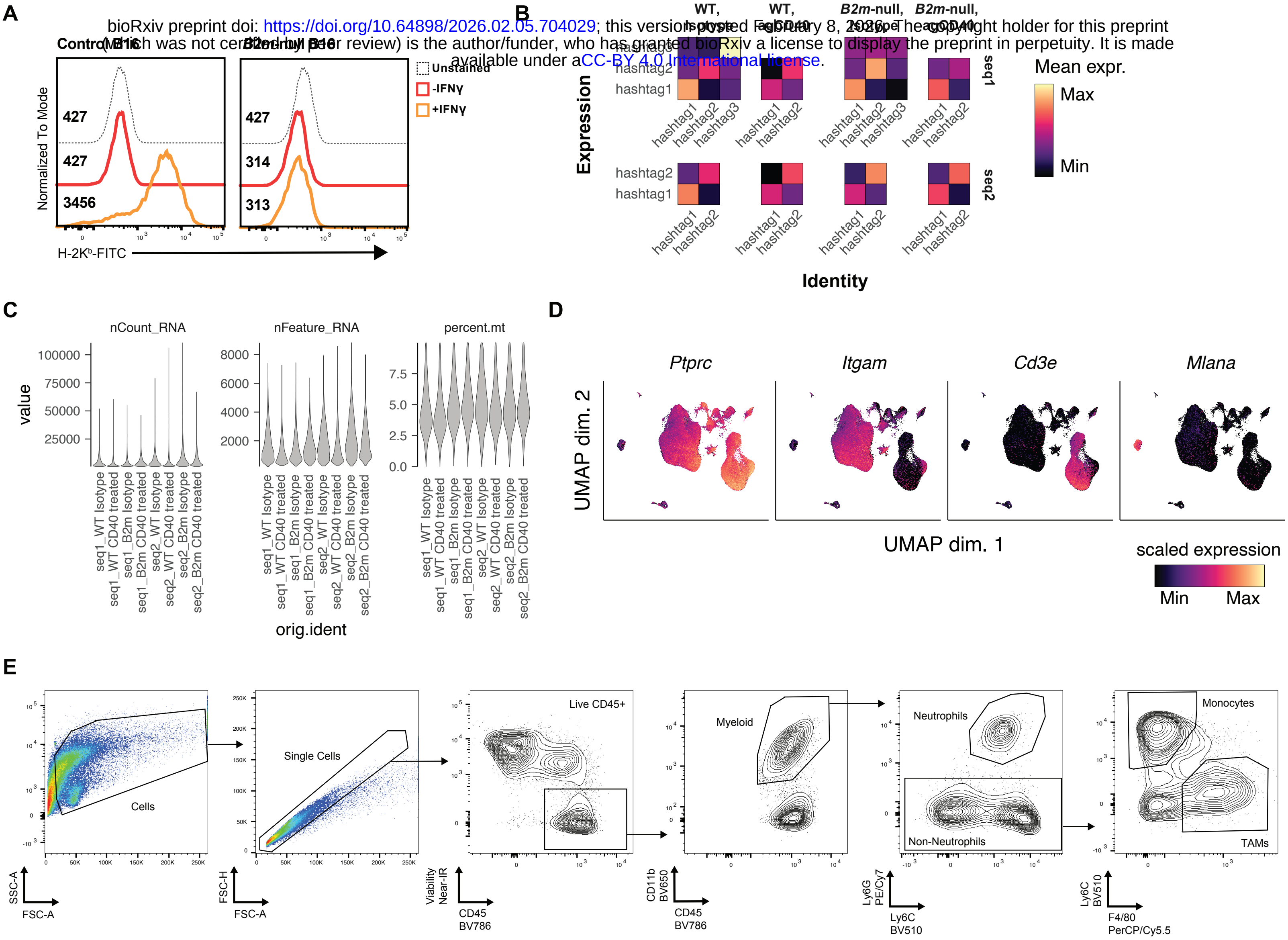

Supplementary Figure 1. (A) Flow cytometry analysis of H-2Kᵇ expression on control or *B2m*-null B16 tumor cells with or without 48 hours IFNγ stimulation. Geometric mean fluorescence intensity shown. Full-minus one (FMO) staining control shown. **(B)** Heatmaps demonstrating average expression of the indicated hashtag sequence within cells assigned to each identity. **(C)** Quality control metrics for the different samples sequenced for single-cell RNA-seq. **(D)** Expression of the indicated genes on the UMAP across all samples and cell types. **(E)** Flow cytometry gating strategy for intratumoral myeloid populations.

Supplementary Figure 2

bioRxiv preprint doi: https://doi.org/10.64898/2026.02.05.704029; this version posted February 8, 2026. The copyright holder for this preprint (which was not certified by peer review) is the author/funder, who has granted bioRxiv a license to display the preprint in perpetuity. It is made available under aCC-BY 4.0 International license.

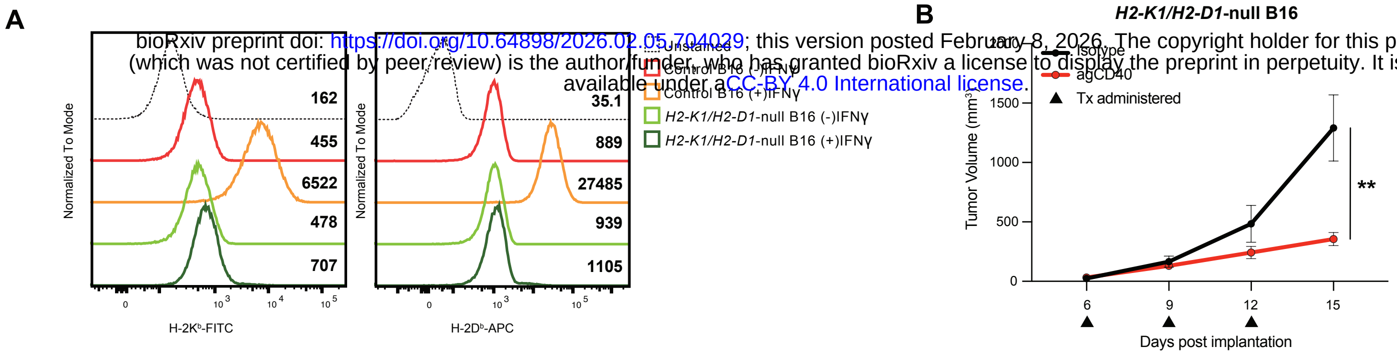

Supplementary Figure 2. (A) Flow cytometry analysis of H-2K^b and H-2D^b expression on control or *H2-K1/H2-D1*-null B16 tumor cells with or without 48 hours IFNγ stimulation. Geometric mean fluorescence intensity shown. Full-minus one (FMO) staining control shown. **(B)** Tumor growth curve of *H2-K1/H2-D1*-null B16 tumor cells implanted into C57BL/6J mice and treated with isotype or agCD40 antibodies on days 6, 9, and 12 post-implantation. ** $p < 0.01$.

Supplementary Figure 3

bioRxiv preprint doi: https://doi.org/10.64898/2026.02.05.704029; this version posted February 8, 2026. The copyright holder for this preprint (which was not certified by peer review) is the author/funder, who has granted bioRxiv a license to display the preprint in perpetuity. It is made available under aCC-BY 4.0 International license.

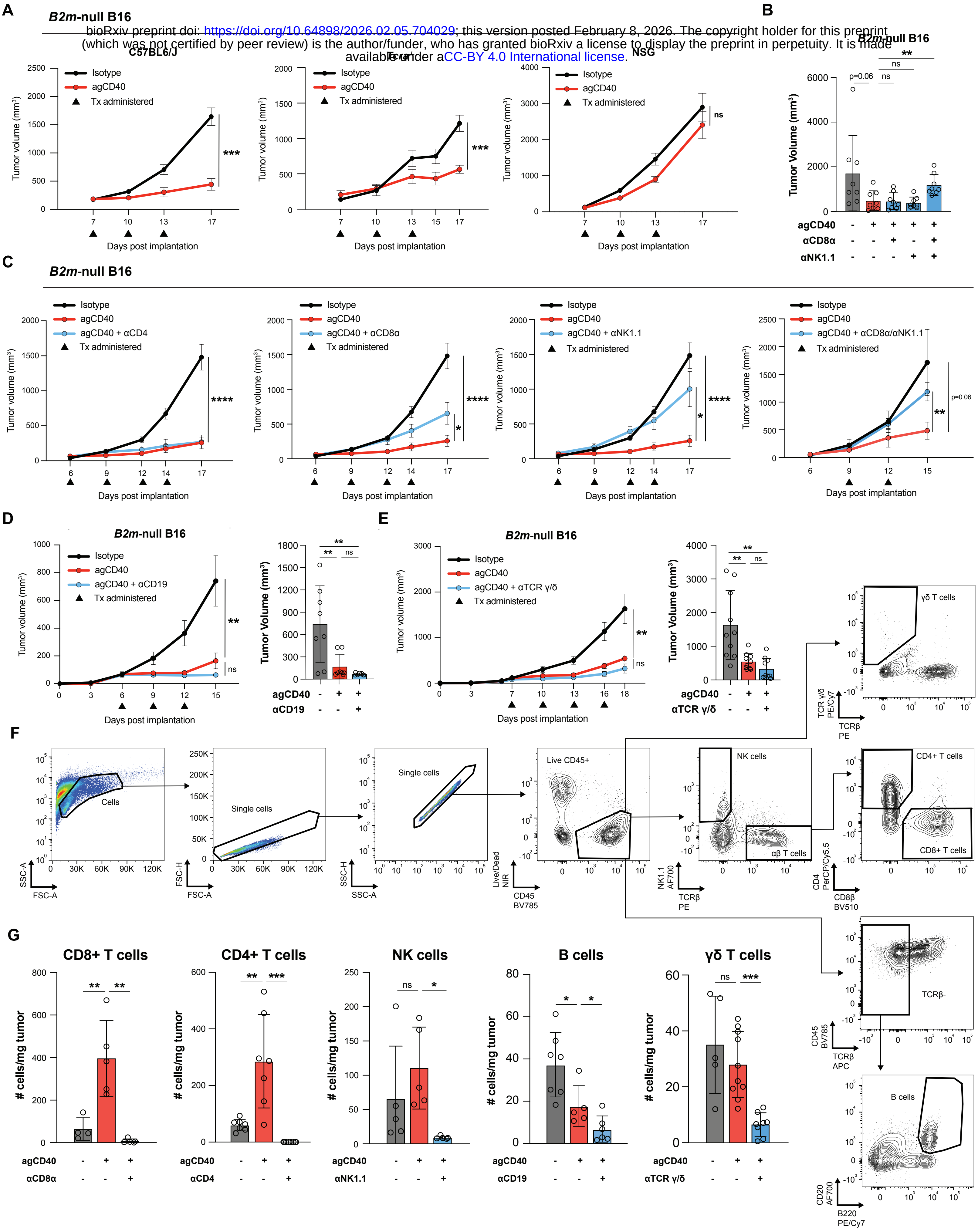

bioRxiv preprint doi: https://doi.org/10.64898/2026.02.05.704029; this version posted February 8, 2026. The copyright holder for this preprint (which was not certified by peer review) is the author/funder, who has granted bioRxiv a license to display the preprint in perpetuity. It is made available under aCC-BY 4.0 International license.

Supplementary Figure 3 (A) Growth curves of *B2m*-null B16 tumors implanted into mice of the indicated genotypes and treated with either CD40 agonist or isotype control antibodies. **(B-E)** Growth curves and final tumor volumes of *B2m*-null B16 tumors implanted into C57BL6/J wild-type mice depleted of the indicated cell types and then treated with CD40 agonist or isotype control antibodies. **(F)** Flow cytometry gating strategy for intratumoral lymphocyte quantification. **(G)** Quantification of the number of immune cells per milligram tumor in the indicated depletion experiments. * $p < 0.05$; ** $p < 0.01$; *** $p < 0.001$; **** $p < 0.0001$; n.s. not significant.

Supplementary Figure 4

bioRxiv preprint doi: https://doi.org/10.64898/2026.02.05.704029; this version posted February 8, 2026. The copyright holder for this preprint (which was not certified by peer review) is the author/funder, who has granted bioRxiv a license to display the preprint in perpetuity. It is made available under aCC-BY 4.0 International license.

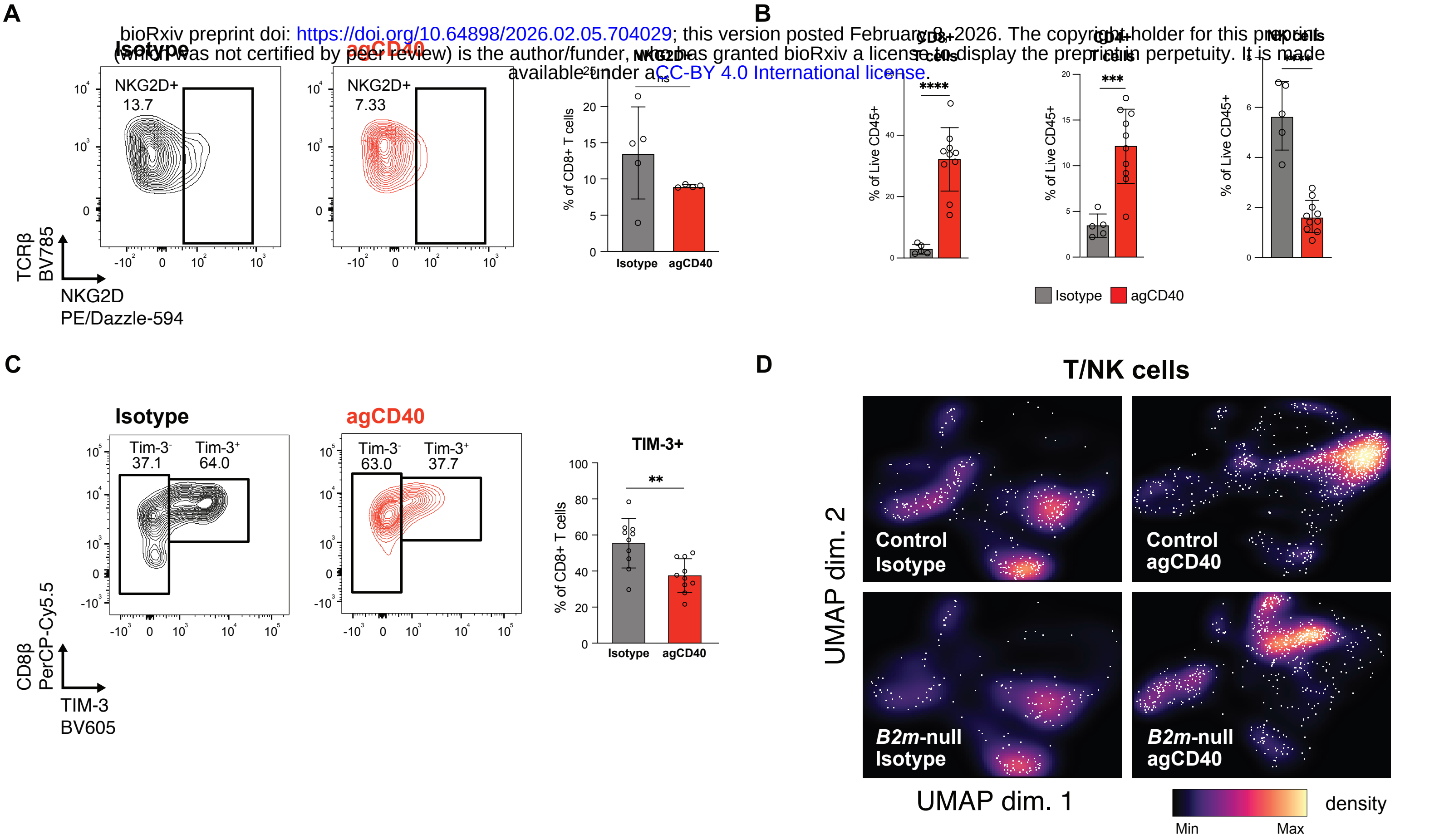

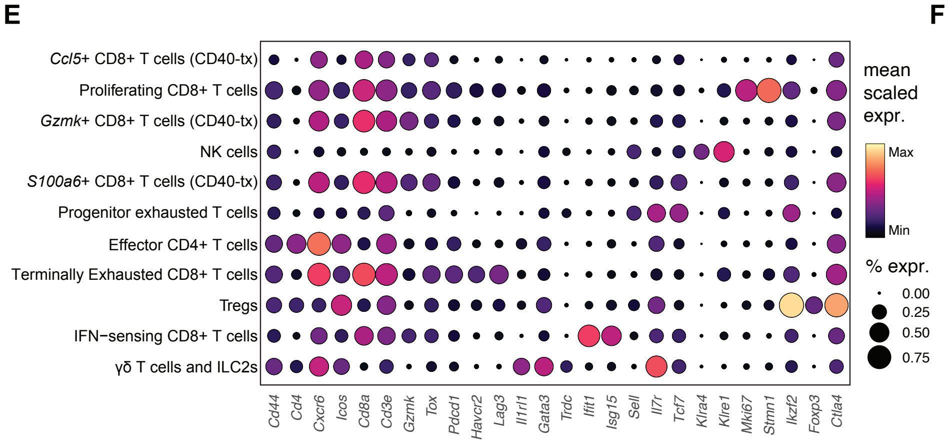

F

cluster	cluster_ID	top_DEG
0	*Ccl5* + CD8+ T cells (CD40-tx)	*Ccl5* , *Hcst* , *Tox* , *Cd8a*
1	Proliferating CD8+ T cells	*Mki67* , *Smnt1* , *Cd8a*
2	*Gzmk* + CD8+ T (CD40-tx)	*Gzmk* , *Ccl5* , *Nkg7* , *Cd8a*
3	NK cells	*Gzma* , *Klra1* , *Klra4* , *Klre1* , *Klrb1c*
4	*S100a6* + CD8+ T cells (CD40-tx)	*Ccl5* , *Hcst* , *S100a6* , *Cd8a*
5	Progenitor exhausted CD8+ T cells	*Il7r* , *Tcf7* , *Satb1* , *Klra1* , *Klra7* , *Jun*
6	Effector CD4+ T cells	*Cd4* , *Icos* , *Cd40lg*
7	Terminally Exhausted CD8+ T cells	*Lag3* , *Havcr2* , *Pdcd1*, *Cd8a*
8	Tregs	*Ikzf2* , *Il2ra* , *Ctla4* , *Foxp3*
9	IFN-sensing CD8+ T cells	*Ifit1* , *Isg15* , *Ifit3* , *Cxcl10* , *Cd8a*
10	γδ T cells and ILC2s	*Il1rl1* , *Gata3* , *Il7r* , *Trdc*

Supplementary Figure 4. (A) NKG2D expression on CD8⁺ T cells from *B2m-null* B16 tumors treated with isotype control (n = 5) or agCD40 (n = 4) antibody. Representative flow plots (left) and quantification (right). **(B)** Flow cytometry quantification of CD8⁺ T cell, CD4⁺ T cell, and NK cell populations as a percentage of Live CD45⁺ cells in *B2m-null* B16 tumors treated with isotype control or agCD40 antibody. Representative results from at least 3 independent experiments. **(C)** TIM-3 expression on CD8⁺ T cells from *B2m-null* B16 tumors treated with isotype control or agCD40 antibody. Representative flow plots (left) and quantification (right) from one of two independent experiments. **(D)** Galaxy plot displaying cell density of T/NK cells subclustered from scRNAseq analysis of control and *B2m-null* B16 tumors treated with agCD40 or isotype-control antibody. **(E)** Dot plot showing indicated mean gene expression (color) and percentage of cells expressing the gene (size) for each of the T/NK cell subclusters. **(F)** Table of T/NK cell subclusters and top differentially expressed genes within each cluster. ** $p < 0.01$; *** $p < 0.001$; **** $p < 0.0001$; n.s. not significant.

Supplementary Figure 5

bioRxiv preprint doi: https://doi.org/10.64898/2026.02.05.704029; this version posted February 8, 2026. The copyright holder for this preprint (which was not certified by peer review) is the author/funder, who has granted bioRxiv a license to display the preprint in perpetuity. It is made available under aCC-BY 4.0 International license.

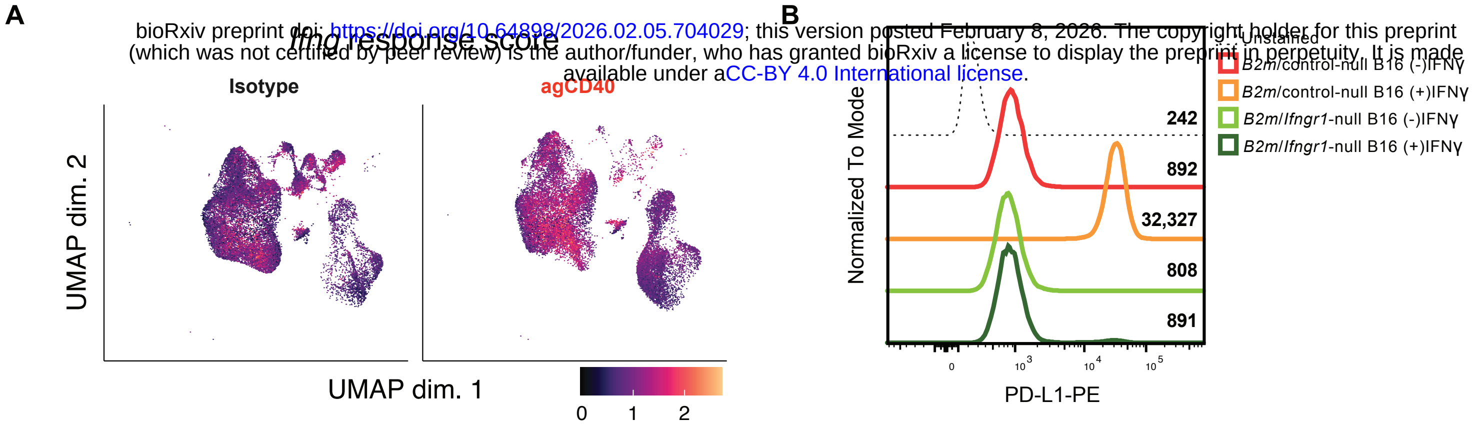

Supplementary Figure 5. (A) Expression of the Hallmark IFNγ response signature on the UMAP showing myeloid cells and T/NK cell clusters. **(B)** Flow cytometry analysis of PD-L1 expression on *B2m-null/control* or *B2m-null/Ifngr1-null* B16 tumor cells with or without 48 hours IFNγ stimulation. Geometric mean fluorescence intensity shown. Full-minus one (FMO) staining control also shown.

bioRxiv preprint doi: https://doi.org/10.64898/2026.02.05.704029; this version posted February 8, 2026. The copyright holder for this preprint (which was not certified by peer review) is the author/funder, who has granted bioRxiv a license to display the preprint in perpetuity. It is made available under aCC-BY 4.0 International license.

Supplementary Figure 6

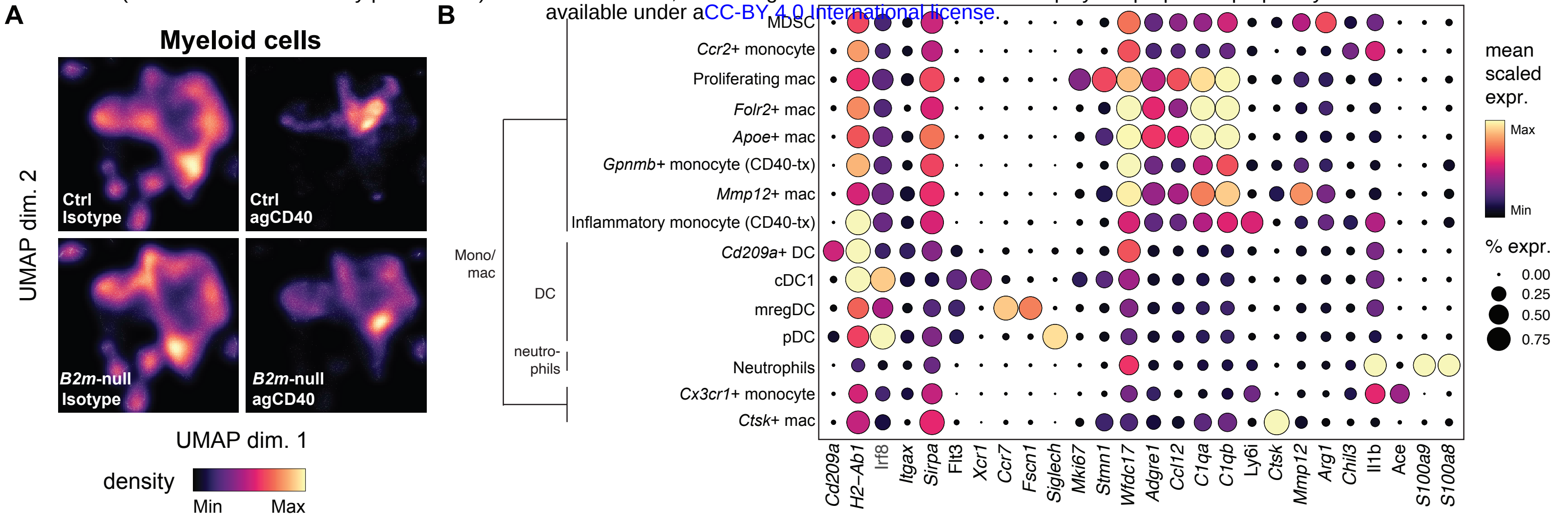

C

cluster	cluster_ID	top_DEG
0	MDSC	*Arg1*, *Spp1*, *Mmp12*
1	*Ccr2* + monocyte	*Ccr2*, *Chil3*, *Il1b*
2	Proliferating macrophage	*Stmn1*, *Top2a*, *Mki67*
3	*Folr2* + macrophage	*Wfdc17*, *Fcgrt*, *Folr2*
4	*Apoe* + macrophage	*C1qa*, *C1qc*, *Apoe*
5	*Gpnmb* + monocyte (CD40-tx)	*Gpnmb*, *AW112010*, *Ctss*, *Creg1*, *Psap*
6	*Mmp12* + mac	*Mmp12*, *Fabp4*, *Fabp5*
7	Inflammatory monocyte (CD40-tx)	*Ly6i*, *Nos2*, *Cxcl9*, *Cxcl10*, *H2-K1*
8	*Cd209a*+ DC	*Cd209a*, *Itgax*, *H2-Ab1*, *Ciita*
9	cDC1	*Irf8*, *Xcr1*, *Itgae*
10	mregDC	*Ccr7*, *Fscn1*, *Cd63*
11	pDC	*Siglech*, *Irf8*, *Ccr9*
12	Neutrophils	*S100a9*, *S100a8*
13	*Cx3cr1* + monocyte	*Itgal*, *Ace*, *Adgre4*, *Nr4a1*
14	*Ctsk* + mac	*Ctsk*, *Mmp9*, *Acp5*, *Dcstamp*

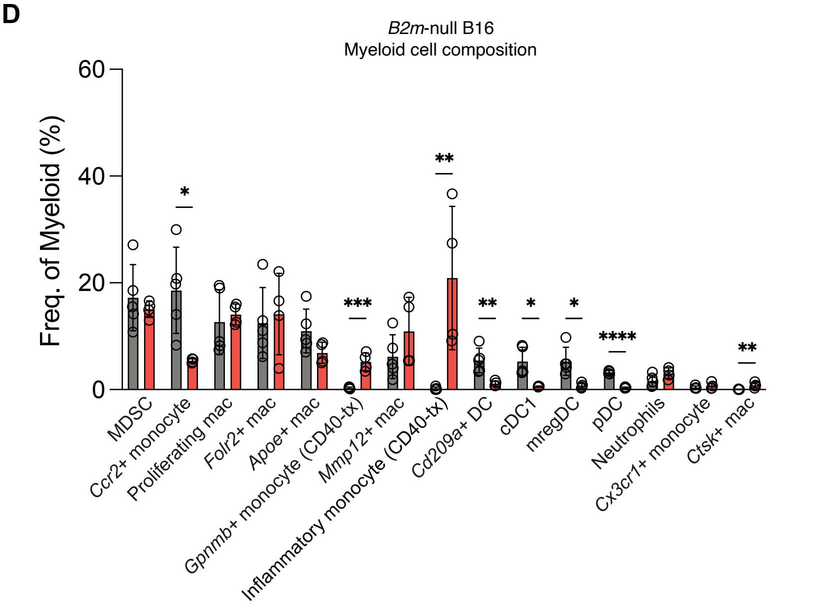

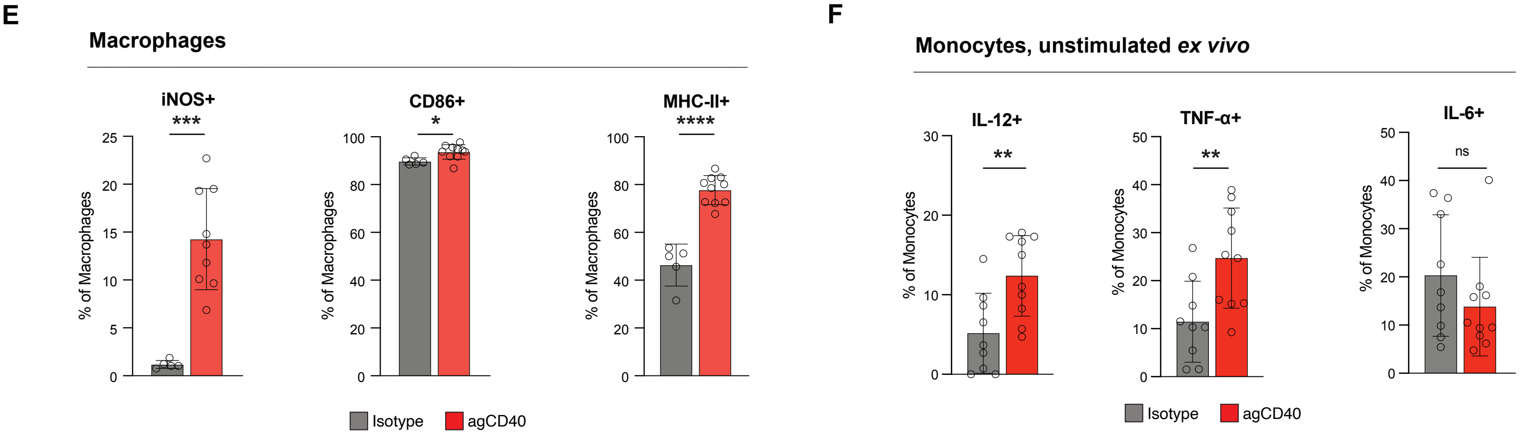

Supplementary Figure 6. (A) Density plot in myeloid cluster UMAP space for each treatment condition. **(B)** Dot plot showing indicated mean gene expression (color) and percentage of cells expressing the gene (size) for each of the myeloid cell subclusters. **(C)** Table of myeloid cell subclusters and top differentially expressed genes within each cluster. **(D)** Quantification of each cell cluster from scRNAseq analysis as a frequency of total immune cells. **(E)** Frequency of iNOS+, CD86+, and MHC-II+ tumor-associated macrophages from isotype control- or agCD40-treated *B2m*-null B16 tumors. **(F)** Frequency of IL-12p40+, TNFα+, and IL-6+ monocytes isolated from isotype control- or agCD40-treated *B2m*-null B16 tumors and plated without stimulation *ex vivo* for 18h. * p <0.05; ** p < 0.01; *** p < 0.001; **** p < 0.0001; ns not significant.

bioRxiv preprint doi: https://doi.org/10.64898/2026.02.05.704029; this version posted February 8, 2026. The copyright holder for this preprint (which was not certified by peer review) is the author/funder, who has granted bioRxiv a license to display the preprint in perpetuity. It is made available under aCC-BY 4.0 International license.

Supplementary Figure 7

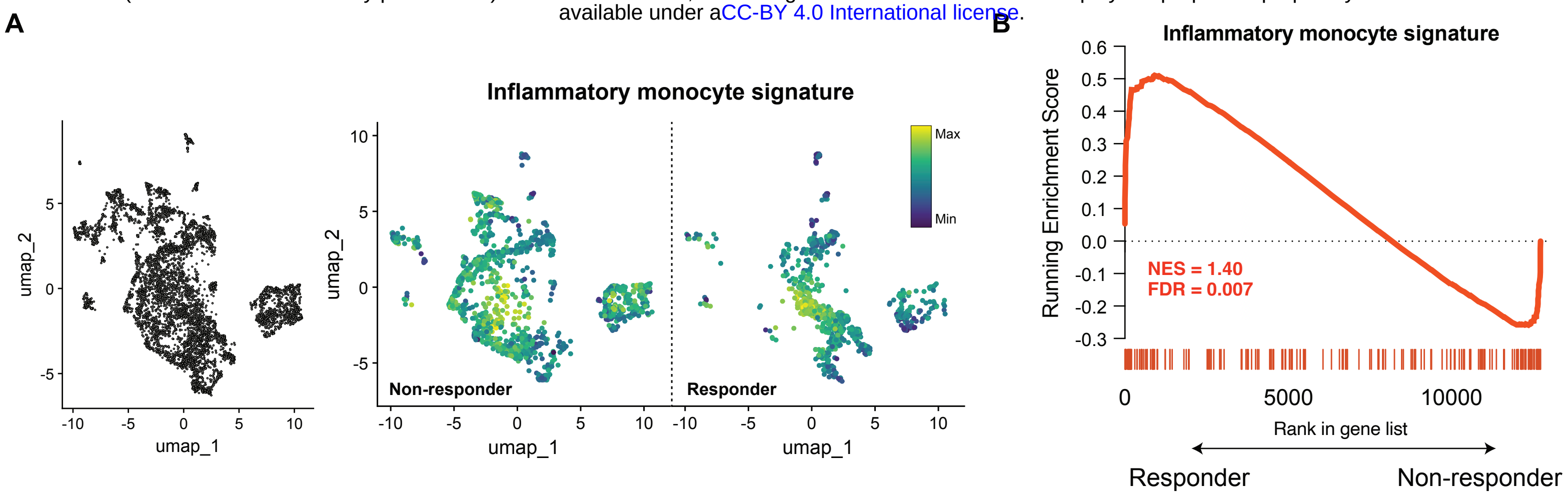

Supplementary Figure 7. (A) UMAP and Inflammatory monocyte signature score in individual myeloid cells (non-responder left, responder right) from human myeloid cell analysis. **(B)** GSEA of the inflammatory monocyte signature in the ranked list of genes differentially expressed in pre-treatment myeloid cells between responders and non-responders. * $p < 0.05$; ** $p < 0.01$; *** $p < 0.001$; **** $p < 0.0001$.
